# Analysis of the foveal microvasculature in sickle cell disease using swept-source optical coherence tomography angiography

**DOI:** 10.1038/s41598-020-68625-8

**Published:** 2020-07-16

**Authors:** A. Mokrane, G. Gazeau, V. Lévy, F. Fajnkuchen, Audrey Giocanti-Aurégan

**Affiliations:** 1 Ophthalmology Department, Avicenne Hospital, 125 Rue de Stalingrad, 93000 Bobigny, France; 2 Clinical Research Unit, Avicenne Hospital, 125 Rue de Stalingrad, 93000 Bobigny, France; 3 Ophthalmology Department, Centre D’Imagerie Et de Laser, 11 rue Antoine Bourdelle, 75015 Paris, France

**Keywords:** Disease genetics, Optical techniques

## Abstract

Ischemic microangiopathy was clearly identified in sickle cell disease (SCD) using fluorescein angiography. A prospective observational clinical study was conducted to assess the foveal avascular zone (FAZ) area and explore perifoveal microvasculature changes in the superficial (SCP) and deep (DCP) capillary plexus using optical coherence tomography angiography (OCTA) and compare two genotypes—HbS/HbS (HbSS) and HbS/HbC (HbSC)-to control. All consecutive patients with electrophoretic confirmation of SCD were included. Swept-source OCTA scans (Triton Plus, Topcon, Tokyo, Japan) with a 3 × 3-mm scanning area and ultra-wide field (UWF) retinography (California, Optos, Fife, Scotland) were recorded for all patients. For OCTA analysis, preset parameters were used to segment the SCP and DCP. The FAZ area was manually assessed. The number of vascular branching points was automatically assessed based on the vascular skeletonization using ImageJ software. Eyes were staged based on Goldberg’s classification of SCD retinopathy (SCDR) using UWF imaging. Forty-six eyes of 24 patients were included in the HbSS (n = 27) and HbSC (n = 19) groups and 16 eyes of 8 unaffected patients in a control group. In the DCP, the FAZ was significantly larger in the HbSC (p = 0.0001) and HbSS (p = 0.0004) groups compared to controls. The FAZ area in the SCP, CRT and number of superficial vascular branching points did not significantly differ between both genotypes. There were less branching points in the HbSC (p = 0.034) and HbSS (p = 0.0014) groups than in controls. The Goldberg stage was significantly higher in the HbSC group than in the HbSS group (2.21 vs. 1.22, p = 0.0062). OCTA provides useful information on macular microvasculature and structural alterations associated with SCDR. Ischemic abnormalities are more predominant in the DCP in case of SCDR and no difference was found between genotypes of patients visually asymptomatic.

## Introduction

Sickle cell disease (SCD) is a common genetic disorder reported worldwide, with a high prevalence in Mediterranean regions, sub-Saharan Africa, Middle East, and Southeast Asia^1^.

This chronic hemolytic disorder is due to the polymerization of a pathologic hemoglobin protein within red cells, resulting into deformed sickle shaped cells and leading to occlusive events and accelerated hemolysis^2^.

SCD includes several genotypes, the most common are homozygous HbS/HbS (HbSS), and heterozygous HbS/HbC (HbSC) or HbS/beta thalassemia^2^.

The most described lesions of sickle cell disease retinopathy (SCDR) are caused by retinal ischemia secondary to the sickling of red blood cells in peripheral retinal vessels^3^. However, macular vessel occlusion due to SCD has also been documented using fluorescein angiography (FA), and histopathological examination^4–6^. These changes include an enlargement of the foveal avascular zone (FAZ), perifoveal capillary non-perfusion, nerve fiber layer infarcts, microaneurysm-like dot and hairpin-shaped venular loops at the posterior pole^4–7^. These microvascular macular changes have also been recently reported with optical coherence tomography angiography (OCTA), an innovative, simple and non-invasive technique that can be used to analyze small retinal vessels^8–11^. Previous studies have shown that heterozygous (HbSC) patients with SCD have a more severe peripheral retinopathy than homozygous (HbSS)^12–14^. However, the distribution of macular lesions between both genotypes has not been assessed yet.

The aim of this study was to assess the FAZ area and explore perifoveal microvasculature changes in the superficial (SCP) and deep (DCP) capillary plexus using OCTA in SCD patients (HbSS and HbSC) and compare both genotypes to unaffected controls.

## Methods

This prospective observational study was conducted in a center specialized in retinal disease imaging and treatment (Ophthalmology department, Avicenne hospital, Paris 13 University, France) in accordance with the Tenets of the Declaration of Helsinki and current French legislation. An informed consent was obtained from all patients. Approval was obtained from the France Macula Federation ethical committee (committee’s reference number: FMF 2018-101137).

All consecutive patients with diagnosed SCD, confirmed by electrophoresis, were examined between June 1st and October 31st 2016, as part of their routine SCD follow-up. All patients were of African descent. We included consecutive patients enrolled when they underwent their annual SCDR surveillance exam or after referral from their hematologist. During the same period, we included the eyes (n = 16) of eight consecutive unaffected African patients matched for age, ethnicity, and axial length in order to limit bias^15^ without ocular pathology, referred for systematic examination.

All patients underwent a complete ophthalmological examination including best-corrected visual acuity (BVCA) assessment using a Monoyer visual acuity chart (results converted into Snellen visual acuity), slit-lamp examination, dilated fundus biomicroscopy, ultra-wide field (UWF) retinography (California, Optos, Fife, Scotland) and OCTA (Triton Plus, Topcon, Tokyo, Japan).

Eyes were staged based on Goldberg’s classification of SCDR using UWF retinography, and UWF angiography when needed^16^.

Two examiners (AM and AGA) analyzed independently UWF retinography and angiography images to stage SCR, based on Goldberg’s classification^16^.

When there was no sign of retinopathy, the stage was 0. Stage I stood for peripheral arteriolar occlusions while Stage II stood for peripheral arteriovenous anastomoses. Stage III corresponded to pre-retinal neovascularization. Finally, intravitreous hemorrhage was classified as stage IV and retinal detachment as stage V.

Inclusion criteria were: age over 18, electrophoretic confirmation of SCD (SS, SC or SB), BCVA ≥ 20/32.

Exclusion criteria were: lens or other ocular media opacities preventing correct imaging, high myopia, diabetes, clinical evidence of any other maculopathy, retinal surgery history, glaucoma, history of macular laser, prior intravitreal injection. OCT-A images with significant image artifacts and poor image quality, including: OCT-A quality score below 60, motion or projection artifacts, blurry images, signal loss or poor centration.

All patients were examined using a 1,050-nm swept-source OCTA instrument (Triton Plus, Topcon, Tokyo, Japan). We performed 3 × 3 mm volume acquisitions (in order to improve image quality in comparison to larger scans), and used the preset parameters to segment the SCP and DCP. For each patient, OCTA of the SCP was obtained with a slab between an inner boundary at 2.6 µm beneath the inner limiting membrane and an outer boundary at 15.6 µm beneath the inner plexiform layer. OCTA images of the DCP were obtained with a slab between the inner and outer boundaries, respectively, at 15.6 and 70.2 µm beneath the inner plexiform layer. All segmentations were controlled and manually segmented if necessary to match each plexus. When a segmentation artifact was detected, we performed a manual segmentation. Raw images were extracted. The motion artifact reduction module was used, and even if the software has a projection artifact removal function^17^, all segmentations were verified for presence of projection artifact which was an exclusion criteria.

### Quantitative analysis

**Figure 1:**
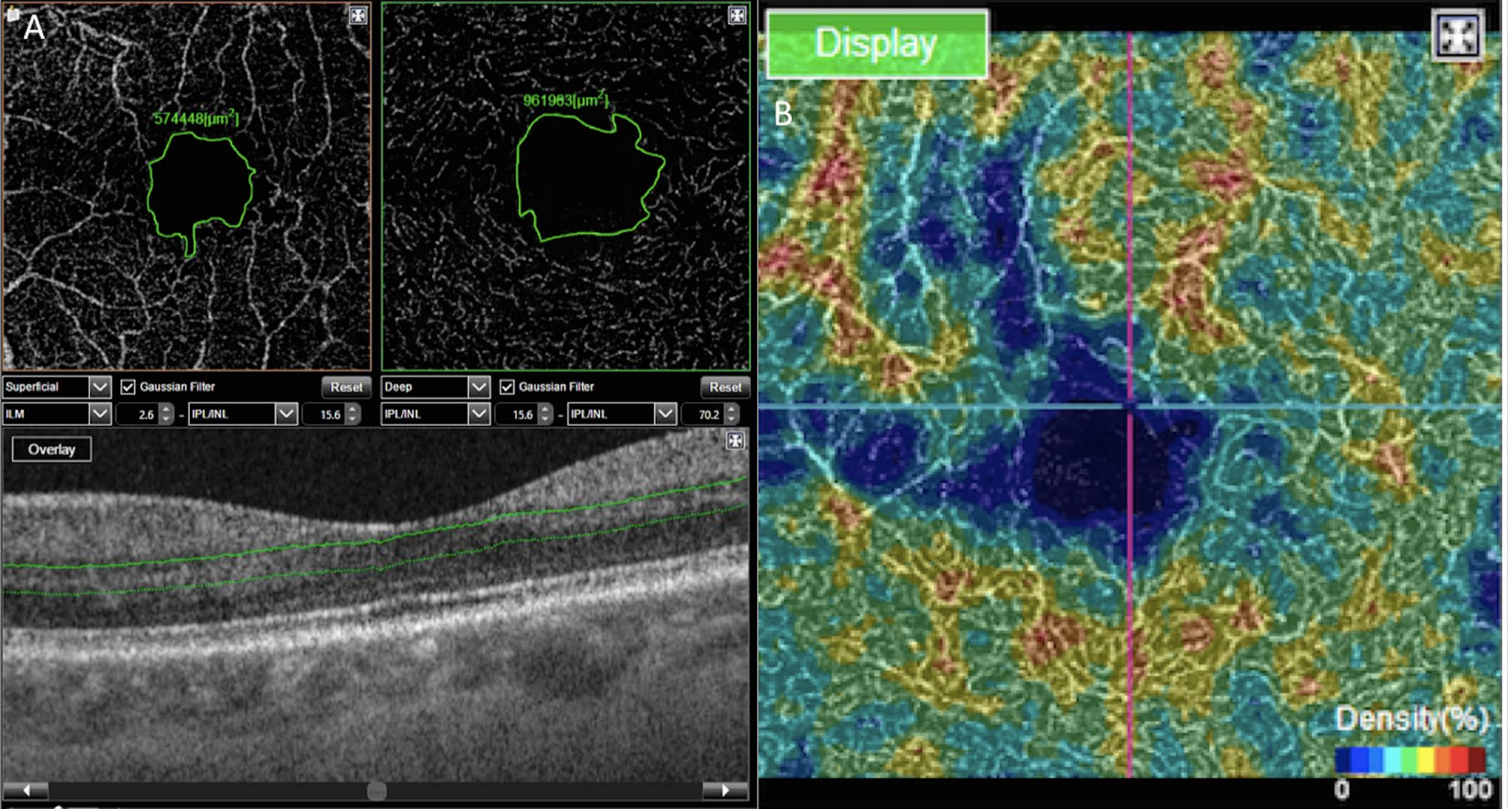
(**A**) Manual measurement of the foveal avascular zone (FAZ). On the left, at the superficial capillary plexus (SCP) level, and on the right, at the deep capillary plexus (DCP) level. The limits of the FAZ are in green. Below, B-scan with the limits of segmentation of the DCP in green. (**B**) Example of colorimetric representation of the vascular density in the DCP used for subjective evaluation of the location of blood flow loss. In blue the lower densities, and in red the higher densities.

The main outcome was the FAZ area (Fig. 1), manually measured using the area measurement tool (in mm^2^) software, drawing the inner wall of the vessels in the SCP and DCP^8,18^. Secondary outcomes were vascular density in the SCP and DCP, and retinopathy severity stage (Goldberg classification).

Two independent examiners (AM and AGA) performed the measures for each eye. In case of disagreement a third observer (FF) adjudicated the result.

To quantify the plexus density, two-dimensional (2D) images were analyzed using Fiji/ImageJ 1.51d (open source image analyzer), on extracted En-Face images.

Image noise was removed by processing a smooth filter (Plugins > Process > Smooth > Gaussian Method—1 sigma). Then, images were binarized (Image > Adjust > Threshold > Apply) and the skeleton was extracted from binary images using Skeletonize (2D/3D) as previously described^19^.

**Figure 2:**
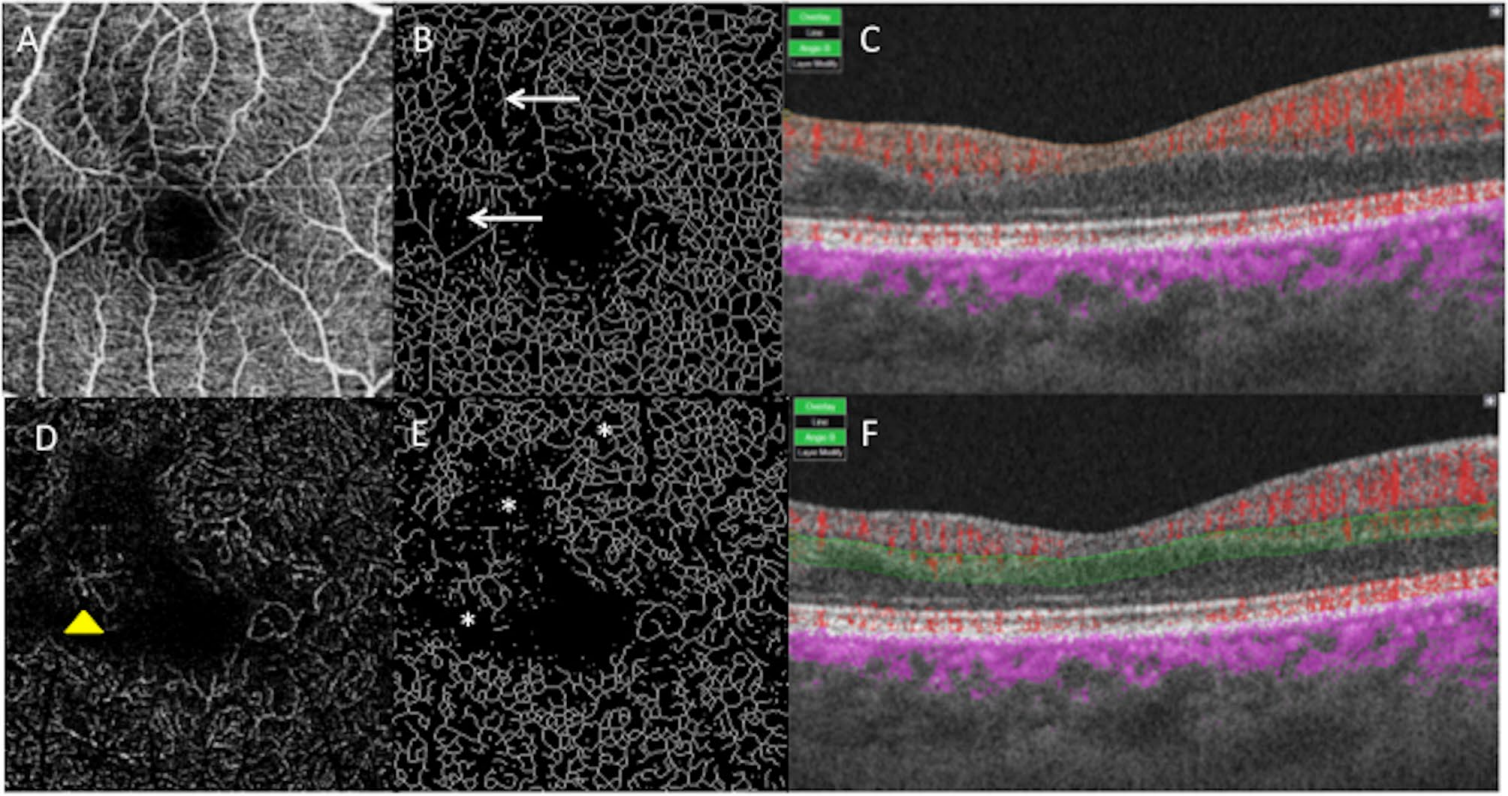
Right eye of a patient with sickle cell disease (SC). (**A**) OCTA of the SCP. (**B**) Image of the SCP obtained after skeletonization. The white arrows are pointing the area of rarefied capillary. (**C**) B-scan showing the limits of segmentation in orange. Blood flow appears in red. (**D**) DCP of the same eye. A dilated capillary is pointed by a yellow arrow-head. (**E**) Image of the DCP obtained after skeletonization. The white stars are localized in the area of rarefied capillary. (**F**) B-scan showing the limits of segmentation in green. Blood flow appears in red.

Finally, the resulting skeletons (Fig. 2) were analyzed on the 2D images using Analyze Skeleton^20^ (2D/3D). For each skeleton, the vascular branching point density was used to compare each plexus density in terms of number of branching points/mm^2^. Since no automatized vascular density quantification was available at the time of the study, with the Triton device, one engineer (GG) designed a custom-made software using Matlab in order to obtain quantification. This software provided a binarization of the raw images in order to assess the vascular density, and a skeletonization of the row image to assess the total vessel length. The size of all images was 232 × 232 pixels. For binarization, the algorithm used the local thresholding function “adaptthresh” embedded in MatLab Image Processing Toolbox. Based on a variant Bradley method^21^, the function performs local thresholding (T = 2 × (sizeimage/16) + 1 corresponding to 30 pixels for size_image_ = 232 or a 0.3 threshold on a 0–1 scale.

### Qualitative analysis

Qualitative abnormalities (Figs. 1 and 2) were identified on the OCTA frame based on the location and distribution of microvasculature abnormalities in the SCP and DCP (temporal, nasal, inferior or superior). A control group included unaffected African patients enrolled on routine examination, without ophthalmological abnormality and without known hemoglobinopathy and they underwent the same examinations as SCD patients. African patients were selected for the control group so that this group was comparable in terms of ethnicity. Morphometric parameters of the fovea depend indeed on ethnicity^22^.

### Statistical analysis

Statistical analyzes were conducted using XLSTAT and PRISM 7, and results were compared using the Mann–Whitney test. In order to assess the reproducibility of the FAZ measurement, we evaluated the intra-class correlation coefficient (ICC) over two measures of each image. ICC was calculated using SPSS statistical package version 20 (SPSS Inc, Chicago, IL) based on a mean-rating assessment (k = 2), consistency-agreement, 2-way random-effects model.

Statistical significance was set at p < 0.05 for all analyses. Data are presented as median (range) or counts (percentage).

## Results

### Patients

**Figure 3:**
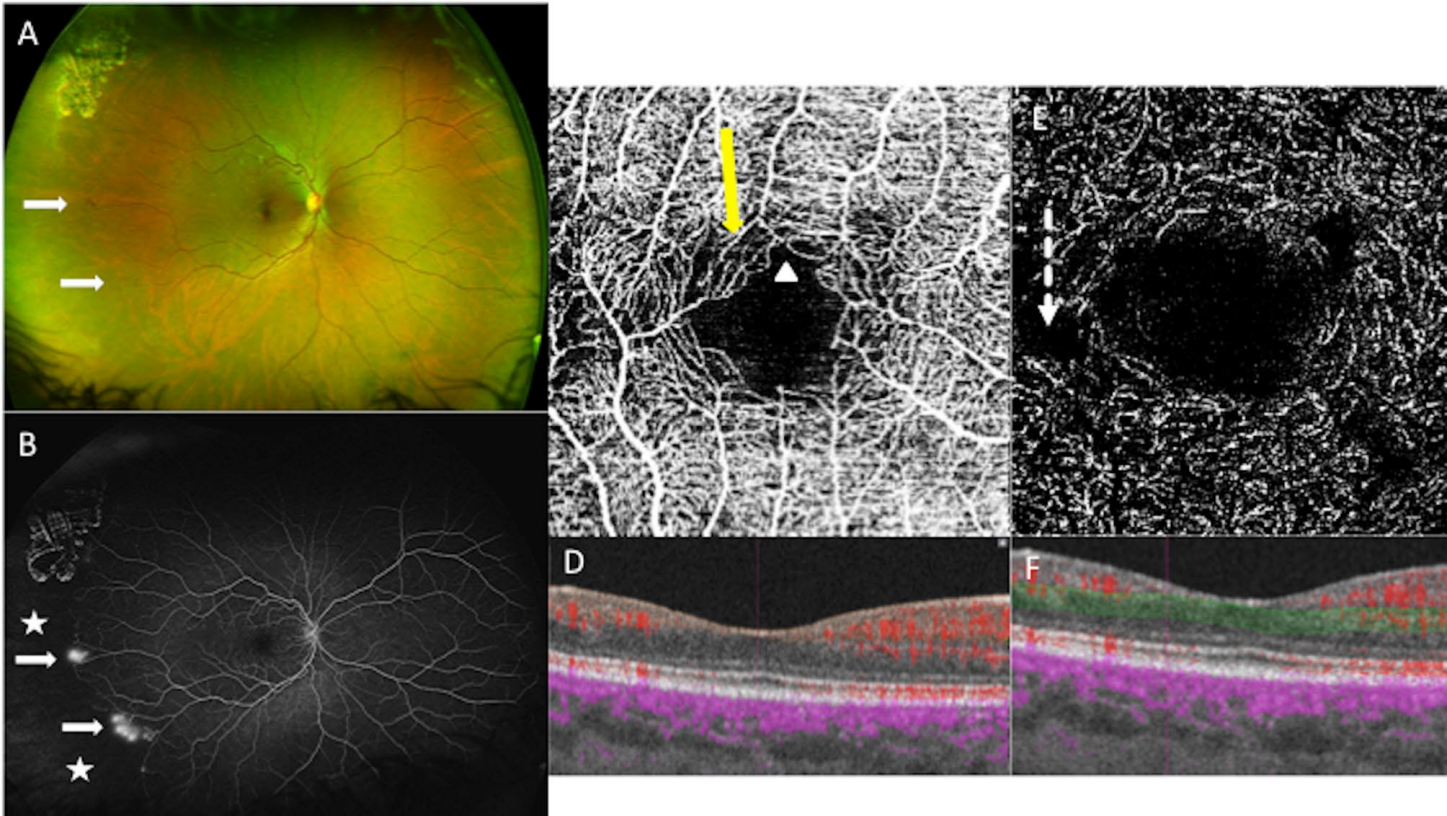
Multimodal imaging of the right eye of a 30-year old woman with Sickle cell retinopathy (SS), stage 3. (**A**) Red/green ultra-wide field retinography with neovascular lesions (white arrows). (**B**) The ultra-wide field fluorescein angiography shows temporal peripheral non-perfusion area (white stars), and preretinal neovascular lesions (white arrows). (**C**) OCT angiography shows microvascular abnormalities in the perifoveal area in the superficial capillary plexus: areas of rarefied capillary (yellow arrow), and disruption of the perifoveal anastomotic capillary arcade (white arrow-head). (**D**) B-scan with the limits in orange of the SCP segmentation. Blood flow appears in red. (**E**) OCT angiography shows microvascular abnormalities in the perifoveal area in the deep capillary plexus: enlargement non-perfusion area (dotted arrow). (**F**) B-scan with the limits in green of the DCP segmentation. Blood flow appears in red.

Forty-six eyes of 24 SCD patients were included in the study. Twenty-seven eyes of 14 homozygous patients (Fig. 3) with SCD (HbSS Group) with a mean age of 33.9 years (min–max: 21–60) were included, and the sex ratio was 4 men/10 women.

Nineteen eyes of ten heterozygous patients with SCD (HbSC Group) with a mean age of 37.8 years (min–max: 32–49) were included and the sex ratio was 4 men/6 women.

All patients in the HbSS and HbSC groups were Africans.

Sixteen unaffected eyes of eight African patients with a mean age of 37.5 years (min–max: 24–49) were included in the control group, and the sex ratio was 1 man/7 women. BCVA was 20/20 in all eyes (SCD and controls). The mean central retinal thickness was 173.8 µm ± 15.7 in the HbSS group, 180 µm ± 14.6 in the HbSC group, and 179.7 µm ± 11.5 in the control group. The main demographic characteristics are presented in Table 1.

**Table 1:** Demographics of patients with sickle cell retinopathy and controls.

	HbSS	HbSC	Controls
Patients/eyes (n)	14/27	10/19	8/16
Age (years)	33.9 ± 11.2	37.8 ± 6.2	37.5 ± 7.6
Men/women (n)	4/10	4/6	1/7
OCT Mean central retina thickness ± SD (µm)	173.8 ± 15.7	180 ± 14.6	179.7 ± 11.5
**UWF imaging stage of retinopathy no. eyes (%)**			
I	18 (66.6)	6 (31.5)	
II	4 (14.8)	1 (5.3)	
III	5 (18.5)	12 (63.2)	
Mean stage ± SD	1.44 ± 0.89	2.31 ± 0.9	

*OCT* optical coherence tomography, *UWF* ultra-wide field.

### Retinopathy severity stage

In the homozygous group (HbSS), stage I was diagnosed in 18 eyes (66.6%), stage II in four eyes (14.8%), stage III in five eyes (18.5%).

In the heterozygous group (HbSC), stage I was diagnosed in six eyes (31.5%), stage II in one eye (5.3%), stage III in 12 eyes (63.2%).

The mean staging was 1.44 and 2.31 (p = 0.0062) in HbSS and HbSC groups, respectively.

### Foveal avascular zone

The mean FAZ area in the SCP was of 0.44 (min–max: 0.1–0.7) mm^2^ in the homozygous group (HbSS), 0.48 (min–max: 0.3–0.7) mm^2^ in the heterozygous group (HbSC) and 0.42 (min–max: 0.2–0.5) mm^2^ in the control group and no statically significant difference was found between groups (p > 0.05, details in Table 2).

**Table 2:** Mean foveal avascular zone (FAZ), vascular branching points and vascular density in the superficial (SCP) and deep (DCP) capillary plexi in the sickle cell disease groups (heterozygous- SC, and homozygous-SS) and control group.

Group n = number of eyes	HbSS n = 27	HbSC n = 19	Control n = 16	HbSS vs control p	HbSC vs control p	HbSC vs HbSS p
Mean superficial FAZ ± SD (range) mm^2^	0.44 ± 0.16 (0.1–0.7)	0.48 ± 0.12 (0.3–0.7)	0.42 ± 0.09 (0.2–0.5)	0.85	0.25	0.49
Mean deep FAZ ± SD (range) mm^2^	0.84 ± 0.3 (0.3–1.5)	0.86 ± 0.28 (0.5–1.5)	0.57 ± 0.09 (0.4–0.8)	**0.0004**	**0.0001**	0.98
Vascular branching points in the SCP (n/mm^2^)	92 ± 21 (49–126)	101 ± 17 (73–131)	98 ± 17 (76–127)	0.29	0.55	0.15
Vascular branching points in the DCP (n/mm^2^)	27.9 ± 5.6 (17–38)	29.9 ± 6.8 (17–41)	37.3 ± 9.4 (27–55)	**0.0014**	**0.034**	0.3
Vascular density in the SCP	0.79 ± 0.01 (0.8–0.9)	0.85 ± 0.01 (0.81–0.87)	0.85 ± 0.01 (0.81–0.89)	**0.01**	0.38	**0.01**
Vascular density in the DCP	0.25 ± 0.02 (0.22–0.3)	0.26 ± 0.01 (0.2–0.29)	0.29 ± 0.02 (0.2–0.35)	**0.0001**	**0.001**	0.4

Bold characters indicate that p-value is significant.

The mean FAZ area in the DCP was significantly larger in the homozygous (HbSS) and heterozygous (HbSC) groups: 0.84 (min–max: 0.3–1.5) mm^2^ and 0.86 (min–max: 0.5–1.5) mm^2^, respectively compared to the control group: 0.57 (min–max: 0.4–0.8) mm^2^ (p = 0.0004 and p = 0.0001, respectively).

ICC was > 0.99 for all groups.

### Vascular density

The vascular density was assessed using two parameters: the number of vascular branching points, which reflects the number of vascular ramifications, and an estimate of the vascular density based on our custom-made software.

Vascular branching points: there was no significant difference in terms of branching point number between all groups in the SCP. There were less branching points in the HbSC (29.9 ± 6.8/mm^2^; p = 0.034) and HbSS (27.9 ± 5.7/mm^2^; p = 0.0014) groups than in the control group (37.3 ± 9.4/mm^2^) (Table 2) in the DCP. No difference was found between the two genotypes.

**Figure 4:**
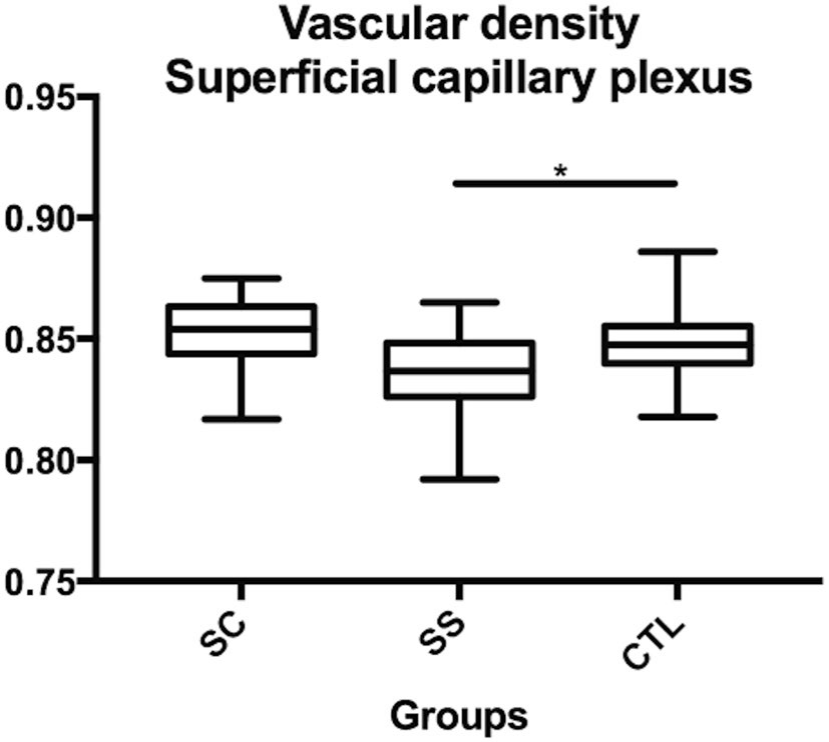
Comparison of the superficial capillary plexus (SCP) vascular density between sickle SC, SS and controls. *p < 0.05.

**Figure 5:**
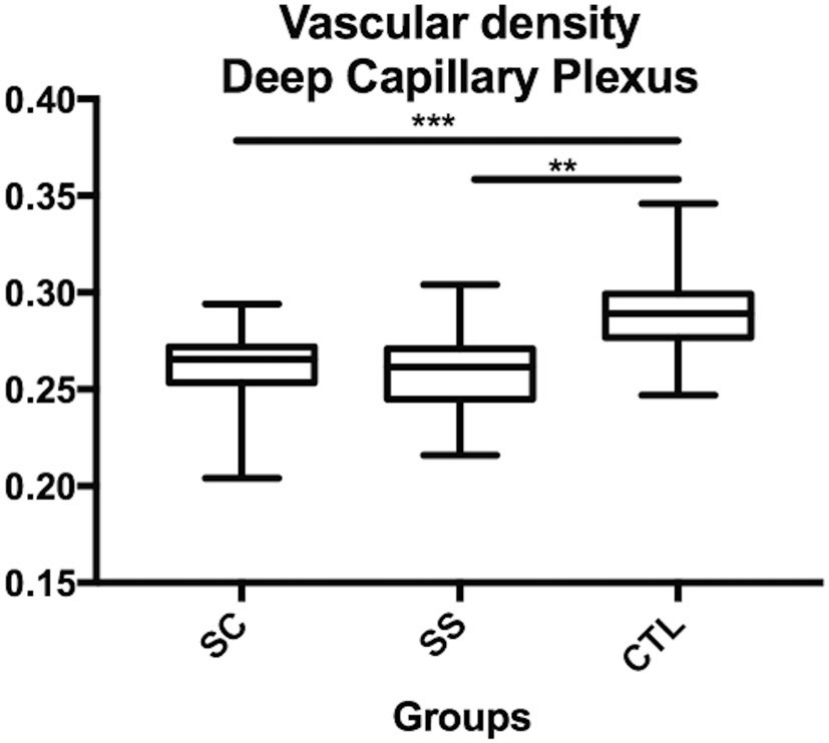
Comparison of the deep capillary plexus (DCP) vascular density between sickle SC, SS and controls. **p < 0.01; ***p < 0.001.

Vascular density: the vascular density was quantified in the whole image (3 × 3 mm), and there was a slightly significant difference between the HbSS group and the control group (p = 0.01) in the SCP (Table 2, Fig. 4). In the DCP (Table 2, Fig. 5), the vascular density was significantly greater in the control group (0.29 ± 0.02) than in the HbSC group (0.26 ± 0.01; p = 0.001) and in the HbSS group (0.25 ± 0.02; p = 0.0001), without significant difference between SS and SC groups. For vascular density, ICC was > 0.99 for all groups.

There was no statistical difference depending on the stage of Goldberg classification, in term of FAZ area, or vascular density. The results are presented in Table 3.

**Table 3:** FAZ area, total vessel length and vascular density of SCP and DCP depending on the stage of Goldberg classification.

	Stage 1	Stage 2	Stage 3	p^p^
**SCP**				
FAZ area (mean)	0.43	0.46	0.48	0.52
Vascular density	0.81	0.84	0.85	0.06
**DCP**				
FAZ area (mean)	0.77	0.94	0.93	0.35
Vascular density	0.26	0.26	0.26	0.5

^p^Kruskal–Wallis Test.

### Qualitative analysis

This analysis is presented in Table 4. All eyes presented with microvascular abnormalities in the perifoveal region of the macula. In the homozygous group (HbSS), we observed a rarefaction of capillaries that appeared dilated in the SCP in 11/27 eyes (41%), and in the DCP in 20/27 eyes (74%) and a disruption of the perifoveal anastomotic capillary arcade of the SCP in 15/27 eyes (55%).

**Table 4:** OCTA qualitative analysis of eyes with sickle cell retinopathy.

	HbSS		HbSC	
	27 eyes		19 eyes	
	SCP	DCP	SCP	DCP
Rarefied and dilated perifoveal capillaries n (%)	11 (41%)	20 (74%)	10 (53%)	16 (84%)
Areas of capillary non-perfusion n (%)	1 (4%)	6 (22%)	1 (5%)	4 (21%)
Disruption of the perifoveal anastomotic capillary arcade n (%)	15 (55%)	NA	14 (74%)	NA

*NA* not applicable.

In the heterozygous group (HbSC), we observed a rarefaction of capillaries that appeared dilated [in the SCP in 10/19 eyes (53%), in the DCP in 16/19 eyes (84%)], and a disruption of the perifoveal anastomotic capillary arcade of the SCP [in 14/19 eyes (74%)].

Microvascular lesions were found predominantly in the DCP in the HbSS and HbSC groups. More than 70% of capillary abnormalities were located in the temporal juxtafoveal region (Fig. 3).

## Discussion

In this study, we report foveal microvasculature changes observed in SCD using swept-source OCTA. We compared our findings between two groups of SCD patients (HbSS and HbSC genotypes) without any vision loss during their standard evaluation.

The detailed imaging of the foveal microvasculature with FA is limited by the attenuation of the luteal pigment and the diffusion of fluorescein that does not allow differentiating the SCP from the DCP, while this is now possible with OCTA. Foveal and perifoveal vascular changes in SCD are known for decades and have been described using FA^4,6,23^. Recently, several studies have reported abnormal macular findings on OCTA images of patients with SCD^8–11^.

Minvielle et al.^8^ have described microvascular abnormalities in the perifoveal area using OCTA imaging in all eyes (Optovue, Avanti), whereas FA appeared normal in 50% of 18 eyes. In this study, mainly including patients with no retinopathy/stage I (52.2%) or II (10.9%), the perifoveal vascular density in the SCP and DCP was significantly reduced in patients with SCD compared to the control group. No distinction was made between the different genotypes of SCD patients. Qualitative vascular changes^8,10,24^ of the SCP and/or DCP have also been described using OCTA in SCD and automated algorithms for computer-aided SCR classification have been developed based on quantitative parameters^25^.

In our study, we found a significantly larger FAZ, less vascular branching points and a lower vascular density in the DCP of SCD patients, compared to the control group. The OCTA findings showed more microvasculature lesions in the DCP, including rarefaction and dilated perifoveal capillaries in 84% of the 19 HbSC eyes in the qualitative analysis (Table 3), while the same parameters (FAZ, vascular branching points) were not or only slightly (vascular density) statistically significant in the SCP. We assumed that since the DCP and the SCP have a different organization and also since it has been recently shown that the DCP is divided into intermediate and deep plexus mainly composed of small size capillaries compared to the SCP, the rarefaction could initially starts in DCP due to the small vessel caliber. Our results on the DCP are consistent with previous studies that have reported macular ischemic changes on OCTA images of patients with SCD^8–10,18,23^.

The absence of significant differences in the SCP between control and SCD patients could be explained by the low severity staging, with 18 (66.6%) HbSS eyes and 6 (31.5%) HbSC eyes of SCD patients with stage I retinopathy, and no stage IV or V in the study, however comparable to previous studies^8^. Also the mean age of our patients was relatively lower (37.5 years) than in similar studies^8^, which could reflect a less advanced disease. Age-dependent linear regression analysis revealed that older patients have larger FAZ^22,26^, when other authors did not find such a correlation^15^. In our series age is comparable in all groups, which is a warrant of the absence of effect of this parameter on our FAZ measures.

Furthermore, more vascular rarefaction of the DCP has been previously reported with OCTA in SCDR, associated with retinal thinning on SD-OCT^8–10^. Tick et al.^22^ have shown that FAZ area correlates inversely with central foveal thickness (CFT) in normal population. In our study, there was no statistical difference in term of CFT between all groups. We have also chose a control group with the same ethnicity than our SCD patients. All our patients (SCD and control) are African like eighty percent of SCD patients^27^ in order to limit the bias.

Goldberg’s classification of SCDR severity has been developed based on the clinical examination and conventional FA imaging^16^. Using this classification, SCD patients with the HbSC genotype have been shown to have a more severe peripheral retinopathy than HbSS genotype^7,14,28^. Our study results confirmed these findings based on UWF retinography in all patients, and angiography when needed. The main strength of our study is the comparison between HbSS and HbSC genotypes in patients with no visual acuity loss, which has not been explored yet in the literature to the best of our knowledge, and showed that both subtypes do not differ in foveal microvasculature abnormalities. This suggests that the mechanisms involved in the peripheral SCDR and in the macular alterations are different. Moreover, we did not find any association between macular vascular density and peripheral non perfusion unlike in vein occlusions^29^, confirming previous results^30^. We can assume that no difference between genotypes is observed, since the DCP, mainly involved in SCD maculopathy, is composed only by vein^31^. At the opposite, at the periphery, arteriolar capillaries could be more affected in HbSC genotypes.

Our study has several limitations however, including the FAZ manual measurements, even when two independent graders analyzed images. Moreover, another limit is the automatic segmentation used by the Triton including both the deep and the intermediate capillary plexus in the DCP segmentation. However our measurement methods were identical in the control and SCD groups. We have assessed the reproducibility of FAZ manual measurements by ICC that shows a good reproducibility between two measures for the same grader and between two graders. Another limitation is the small size of our cohort but comparable to most of the studies^8,10^. Despite the size of our sample, the mean FAZ area was significantly different in the DCP of SCD patients than in control patients, and the Goldberg score was higher in HbSC than in HbSS group and this is consistent with the literature. Finally, we chose 3X3 mm scans, eliminating one of the advantages of SS-OCT. However, we have used the first generation of SS-OCT by Topcon for OCTA, and the quality of the images was not sufficient for analysis with larger scans than 3 × 3 mm at the time of the study.

Finally, our evaluation of the vascular density is also a limitation. We used swept-source OCTA, Triton, that did not provide an automatic measurement of the retinal vascular density at the time of the study. Even if our automatized custom-made software allowed reproducible repeated measurements, it is not as accurate as software provided by OCT companies.

In conclusion, Swept-source OCTA provides useful information on macular microvasculature and structural alterations associated with SCDR. However, the clinical significance of these lesions remains to be established. This study highlights the absence of difference between genotypes in term of fovea microvasculature abnormalities in absence of visual loss conversely to the peripheral SCDR, which is more commonly observed in the HbSC genotype. Further studies with correlation of OCTA, UWF imaging, and function testing such as microperimetry, could help to characterize SCD maculopathy.

## References

[1] Stuart MJ, Nagel RL (2004). Sickle-cell disease. Lancet Lond. Engl..

[2] Emerson, G.G., *et al*. Hemoglobinopathies A2. In (eds. Ryan, S.J., Hinton, D.R., *et al*.) 1429–1445 (2006).

[3] Sayag D (2008). Retinal photocoagulation for proliferative sickle cell retinopathy: A prospective clinical trial with new sea fan classification. Eur. J. Ophthalmol..

[4] Acacio I, Goldberg MF (1973). Peripapillary and macular vessel occlusions in sickle cell anemia. Am. J. Ophthalmol..

[5] Romayanada N, Goldberg MF, Green WR (1973). Histopathology of sickle cell retinopathy. Trans. Am. Acad. Ophthalmol. Otolaryngol..

[6] Asdourian GK (1976). Macular and perimacular vascular remodelling sickling haemoglobinopathies. Br. J. Ophthalmol..

[7] Stevens TS (1974). Sickling hemoglobinopathies; macular and perimacular vascular abnormalities. Arch. Ophthalmol. Chic. Ill.

[8] Minvielle W (2016). Macular microangiopathy in sickle cell disease using optical coherence tomography angiography. Am. J. Ophthalmol..

[9] Falavarjani KG (2016). Correlation of multimodal imaging in sickle cell retinopathy. Retina Phila. Pa.

[10] Han IC, Tadarati M, Scott AW (2015). Macular vascular abnormalities identified by optical coherence tomographic angiography in patients with sickle cell disease. JAMA Ophthalmol..

[11] Han IC, Tadarati M, Pacheco KD, Scott AW (2017). Evaluation of macular vascular abnormalities identified by optical coherence tomography angiography in sickle cell disease. Am. J. Ophthalmol..

[12] Fox PD, Dunn DT, Morris JS, Serjeant GR (1990). Risk factors for proliferative sickle retinopathy. Br. J. Ophthalmol..

[13] Downes SM (2005). Incidence and natural history of proliferative sickle cell retinopathy: Observations from a cohort study. Ophthalmology.

[14] Leveziel N (2012). Sickle-cell retinopathy: Retrospective study of 730 patients followed in a referral center. J. Fr. Ophtalmol..

[15] Linderman R (2017). Assessing the accuracy of foveal avascular zone measurements using optical coherence tomography angiography: segmentation and scaling. Transl. Vis. Sci. Technol..

[16] Goldberg MF (1971). Classification and pathogenesis of proliferative sickle retinopathy. Am. J. Ophthalmol..

[17] Munk MR (2017). OCT-angiography: A qualitative and quantitative comparison of 4 OCT-A devices. PLoS ONE.

[18] Lee CM (1987). Quantification of macular ischaemia in sickle cell retinopathy. Br. J. Ophthalmol..

[19] Giocanti-Auregan A (2016). Altered astrocyte morphology and vascular development in dystrophin-Dp71-null mice. Glia.

[20] Arganda-Carreras I, Fernández-González R, Muñoz-Barrutia A, Ortiz-De-Solorzano C (2010). 3D reconstruction of histological sections: Application to mammary gland tissue. Microsc. Res. Tech..

[21] Bradley D, Roth G (2007). Adaptive thresholding using the integral image. J. Graph Tools.

[22] Tick S (2011). Foveal shape and structure in a normal population. Invest. Ophthalmol. Vis. Sci..

[23] Sanders RJ, Brown GC, Rosenstein RB, Magargal L (1991). Foveal avascular zone diameter and sickle cell disease. Arch. Ophthalmol. Chic. Ill.

[24] Sanfilippo CJ, Klufas MA, Sarraf D, Tsui I (2015). Optical coherence tomography angiography of sickle cell maculopathy. Retin. Cases Brief Rep..

[25] Alam M, Thapa D, Lim JI, Cao D, Yao X (2017). Computer-aided classification of sickle cell retinopathy using quantitative features in optical coherence tomography angiography. Biomed. Opt. Express.

[26] Iafe NA, Phasukkijwatana N, Chen X, Sarraf D (2016). Retinal capillary density and foveal avascular zone area are age-dependent: Quantitative analysis using optical coherence tomography angiography. Invest. Ophthalmol. Vis. Sci..

[27] Ware RE, de Montalembert M, Tshilolo L, Abboud MR (2017). Sickle cell disease. Lancet Lond. Engl..

[28] Ryan SJ (1974). Occlusion of the macular capillaries in sickle cell hemoglobin C disease. Am. J. Ophthalmol..

[29] Seknazi D (2018). Optical coherence tomography angiography in retinal vein occlusion: Correlations between macular vascular density, visual acuity, and peripheral nonperfusion area on fluorescein angiography. Retina Phila. Pa.

[30] Lynch G (2019). Foveal avascular zone morphology and parafoveal capillary perfusion in sickle cell retinopathy. Br. J. Ophthalmol..

[31] Bonnin, S. *et al*. New insight into the macular deep vascular plexus imaged by optical coherence tomography angiography. *Retina (Philadelphia, Pa.)* **35**(11), 2347–2352 (2015). 10.1097/IAE.0000000000000839 26469532

